# Identifying environmental sounds: a multimodal mapping study

**DOI:** 10.3389/fnhum.2015.00567

**Published:** 2015-10-21

**Authors:** Barbara Tomasino, Cinzia Canderan, Dario Marin, Marta Maieron, Michele Gremese, Serena D'Agostini, Franco Fabbro, Miran Skrap

**Affiliations:** ^1^Istituto di Ricovero e Cura a Carattere Scientifico “E. Medea”, Polo Regionale del Friuli Venezia GiuliaUdine, Italy; ^2^Fisica Medica A.O.S. Maria della MisericordiaUdine, Italy; ^3^Unità Operativa di Neuroradiologia, A.O.S. Maria della MisericordiaUdine, Italy; ^4^Unità Operativa di Neurochirurgia, A.O.S. Maria della MisericordiaUdine, Italy

**Keywords:** environmental sounds, fMRI, neurosurgical patients, Activation Likelihood Estimation (ALE) meta-analysis, lesion mapping

## Abstract

Our environment is full of auditory events such as warnings or hazards, and their correct recognition is essential. We explored environmental sounds (ES) recognition in a series of studies. In study 1 we performed an Activation Likelihood Estimation (ALE) meta-analysis of neuroimaging experiments addressing ES processing to delineate the network of areas consistently involved in ES processing. Areas consistently activated in the ALE meta-analysis were the STG/MTG, insula/rolandic operculum, parahippocampal gyrus and inferior frontal gyrus bilaterally. Some of these areas truly reflect ES processing, whereas others are related to design choices, e.g., type of task, type of control condition, type of stimulus. In study 2 we report on 7 neurosurgical patients with lesions involving the areas which were found to be activated by the ALE meta-analysis. We tested their ES recognition abilities and found an impairment of ES recognition. These results indicate that deficits of ES recognition do not exclusively reflect lesions to the right or to the left hemisphere but both hemispheres are involved. The most frequently lesioned area is the hippocampus/insula/STG. We made sure that any impairment in ES recognition would not be related to language problems, but reflect impaired ES processing. In study 3 we carried out an fMRI study on patients (vs. healthy controls) to investigate how the areas involved in ES might be functionally deregulated because of a lesion. The fMRI evidenced that controls activated the right IFG, the STG bilaterally and the left insula. We applied a multimodal mapping approach and found that, although the meta-analysis showed that part of the left and right STG/MTG activation during ES processing might in part be related to design choices, this area was one of the most frequently lesioned areas in our patients, thus highlighting its causal role in ES processing. We found that the ROIs we drew on the two clusters of activation found in the left and in the right STG overlapped with the lesions of at least 4 out of the 7 patients' lesions, indicating that the lack of STG activation found for patients is related to brain damage and is crucial for explaining the ES deficit.

## Introduction

Sound recognition such as a telephone ringing or a dog barking seems such an effortless task. The ability to process environmental sounds (ES) such as warnings (e.g., a siren), threats (e.g., a rattlesnake), recognize when a device is functioning correctly (e.g., clicking of a stapler) or incorrectly (e.g., water dripping), locate an event in space (e.g., an explosion), monitor a change in status (e.g., chiming of a cuckoo clock), communicate an emotional (e.g., scream), or physical condition (e.g., a burp) (Marcell et al., 2000) is essential for everyday life.

The impaired capacity to recognize sounds despite adequate speech comprehension and hearing is defined as auditory agnosia. Auditory agnosia is a rare neuropsychological disorder; the literature about auditory agnosia mainly consists of case studies. The lesions related to this disorder are not particularly consistent as they can involve the temporal or temporo-parietal cortex (Vignolo, 1982; Fujii et al., 1990; Schnider et al., 1994; Engelien et al., 1995; Clarke et al., 2000, 2002; Saygin et al., 2003), subcortical areas (Kazui et al., 1990) such as the thalamus (Clarke et al., 2000) as well as the putamen (Taniwaki et al., 2000), the right hemisphere (e.g., Vignolo, 1982; Fujii et al., 1990; Schnider et al., 1994; Clarke et al., 1996), the left hemisphere (e.g., Vignolo, 1982; Schnider et al., 1994; Clarke et al., 1996, 2000), and both hemispheres (Rosati et al., 1982; Vignolo, 1982; Mendez and Geehan, 1988; Engelien et al., 1995; Clarke et al., 1996; Nové-Josserand et al., 1998). Left hemisphere (and bilateral) lesions tend to produce additional deficits in verbal comprehension. Thus, so far no precise anatomical locations have been correlated with auditory agnosia (Lewis et al., 2004) and knowledge of the brain regions and processing pathways that make up the non-verbal sound recognition system is still fragmentary (Lewis et al., 2004) as many regions have been found to be involved in such processing.

The network of areas involved in ES processing has been investigated also by functional imaging studies (see, for example, the ES processing model by Lewis et al., 2004). In particular, activations in the posterior middle temporal gyrus (MTG) as well as areas like the inferior frontal gyrus (IFG) (Lewis et al., 2004), which is anatomically connected with the auditory cortex (Hackett et al., 1999; Romanski et al., 1999a,b; Romanski and Goldman-Rakic, 2002) have been reported. Areas of activation were found in the MTG and the precuneus bilaterally and in the posterior portion of the left IFG—activation in this area was higher for sound recognition vs. sound localization (Maeder et al., 2001). In addition, activation can be found in the insula (Sharda and Singh, 2012), an area which has numerous direct connections with the auditory cortex (Bamiou et al., 2003) and can cause auditory agnosia if lesioned bilaterally (Engelien et al., 1995). Also the parahippocampal gyri (Sharda and Singh, 2012) can be activated by sound recognition, possibly reflecting the “imageability” of ES sounds (Engel et al., 2009). Lastly, sound activations were found in various subcortical regions like the thalamus (Sharda and Singh, 2012)—which is part of the auditory pathway—as well as the caudate and putamen. Some studies suggested that activation is rather right lateralized, especially in the (non-primary) auditory cortex such as the superior temporal gyrus (STG) (Bergerbest et al., 2004) and the inferior prefrontal cortex (Bergerbest et al., 2004). A PET study (Zatorre et al., 1992) showed that cerebral blood flow (CBF) in the inferior prefrontal cortex depends on the type of cognitive operation involved in ES, see also (Specht and Reul, 2003). In a similar vein, some authors (Thierry et al., 2003) proposed that the connectivity to the left lateralized semantic network is primarily right-sided for ES and left-sided for words. Others (Specht and Reul, 2003) argued that activation in the right STG and superior temporal sulcus (STS) plays the same crucial role in the analysis of non-speech sounds as does the left STS in speech perception (Specht and Reul, 2003). Last, Dick et al. (2007) found that language and ES stimuli evoked very similar volumes of activation in their language-based regions of interest in the left hemisphere, whereas they found greater activation for ES stimuli in the right hemisphere. Studies also showed that the areas involved in ES processing can be modulated by type of stimulus. Like semantic processing, ES recognition is characterized by category specificity: vocalizations (Fecteau et al., 2004; Lewis et al., 2009; Rauschecker and Scott, 2009; Staeren et al., 2009; Leaver and Rauschecker, 2010) and human-produced action sounds (Lewis, 2006; Lewis et al., 2006; Altmann et al., 2007) are different classes of stimuli triggering different activations. To sum up, inconsistencies as to the areas involved in the network supporting ES processing are found across studies, similarly to what emerges from the analysis of the neuropsychological data reported above.

Considering these discrepancies, the aim of our study was to investigate which nodes of the network triggered by ES processing are essential for ES processing and which areas are accessory. We thus compared neuroimaging and neuropsychological data between ES patients and normal controls. In addition, being a correlation-based method, fMRI can delineate brain networks engaged in ES processing; critical mechanisms can only be reported by studying patients with a deficit in ES following a brain damage. We first performed an Activation Likelihood Estimation (ALE) Meta-Analysis to investigate the regions which were found to be consistently activated in neuroimaging studies of ES recognition. In study 2 we selected neurosurgical patients with lesions involving these areas and tested their ES abilities. In study 3 we performed an fMRI study to understand how the key areas involved in ES recognition are functionally deregulated as compared to those of control subjects. In our patients we investigated which parts of the network involved in ES processing that were found to be activated by the meta-analysis of fMRI studies are critically involved in the task. We also tested which part of the ES processing network which was found to be activated by the meta-analysis would be deregulated in the patients' fMRI maps. We acknowledge that our sample size is relatively small, however it is known that auditory agnosia for ES is a rare neuropsychological disorder. Previously published neuropsychological reports focused mainly on single cases, with few exceptions of group studies. Furthermore, at variance with previous studies, our patient sample had selective and relatively small lesions in comparison to patients with stroke lesions which typically involve large parts of the cortex or to patients with neurodegenerative disorders which affect multiple areas.

## Materials and methods

We performed three consecutive studies. In Study 1 we used an Activation Likelihood Estimation (ALE) Meta-Analysis to identify the areas which are consistently activated in neuroimaging studies of ES processing. In Study 2, we report on neurosurgical patients who had a lesion involving the areas revealed by the ALE Meta-Analysis and were impaired at ES recognition. In Study 3 we compared the fMRI maps of patients and healthy controls to understand how these areas might be functionally deregulated because of the lesion.

### Study 1: Activation likelihood estimation (ALE) meta-analysis of neuroimaging studies of environmental sound recognition

#### Data used for the meta-analysis

The functional imaging studies included in this meta-analysis were obtained from a comprehensive PubMed, ISI web of knowledge and Cochrane database literature review focusing on ES recognition (search strings: “ES,” “fMRI,” “PET,” “SPECT”). The references of the retrieved articles were screened in order to identify additional articles dealing with the neural correlates of ES recognition. Inclusion criteria were as follows: neurologically healthy adults and experiments requiring participants to process ES during fMRI/PET/SPECT measurements. The foci employed in each study had to be reported in a standard reference space (Talairach/Tournoux, MNI). Differences in coordinate spaces (MNI vs. Talairach space) were accounted for by transforming coordinates reported in Talairach space into MNI coordinates using a linear transformation model (Lancaster et al., 2007). A random-effects analysis was performed, and single-subject reports were excluded. Table 1 reports the significance level of the reported activations. All studies reported activations surviving corrections for multiple comparisons except some (study 2, 16, 19, 20, 22, 27, and 37).

**Table 1:** **Publications included in the meta-analysis**.

**Experiment N**.	**First Author**	**Year**	**Reference**	**Stimuli**	**Control task in the experimental run**	**Task**	**Silent listening (L) or judgment (J)**	**Button press**	**N Part**	**Scanner**	**Rest/silence (R-S) as control in the contrast image**	**Contrast**	**Threshold**	**Foci**
1	Leech	2011	B&L	E (machine, human, event, alarm, animal, music, vehicle), S	Silent events	Listen carefully and try to understand each sound	L	–	7	3T	R	E > rest	Corr M.C. *P* < 0.0001	3
2	Kraut	2006	JoCN	E (A and living Obj)	Ml	Decide whether they perceived a real, familiar or non-real unfamiliar sound	L	V	18	1.5T		A > non living (Obj)	*P* < 0.001 uncorr	8
3	Kaplan	2007	Cogn Process	Act (hands tearing a piece of paper)	Control sounds	Listening	J	–	10	3T		Act > ctr sounds	*Z* > 2.3 corrected (cluster) significant threshold of *p* = 0.01	11
4	Hashimoto	2006	Neuroimage	A	Pure noise Pure tones	Classified these sounds into birds and animals or pure tones or pure noise	J	V	26	1.5T		A > ctr	*p* = 0.05 corrected for M.C.	3
5	Engel	2009	Neuroimage	H, A, M, E	Silent events	Determine silently whether or not a human was directly involved with the production of the action sound	J	V	20	3T		H > AME	Whole brain correction at α < 0.05	16
6	Engel	2009	Neuroimage	H, A, M, E	Silent events	Determine silently whether or not a human was directly involved with the production of the action sound	J	V	20	3T		A > HME	Whole brain correction at α < 0.05	3
7	Engel	2009	Neuroimage	H, A, M, E	Silent events	Determine silently whether or not a human was directly involved with the production of the action sound	J	V	20	3T		M > HAE	Whole brain correction at α < 0.05	3
8	Engel	2009	Neuroimage	H, A, M, E	Silent events	Determine silently whether or not a human was directly involved with the production of the action sound	J	V	20	3T		E > HAM	Whole brain correction at α < 0.05	4
9	Dick	2007	JoCN	E	Tones	Match sound to picture and as control match identical or different tones to identical or different shapes	J	V	12	1.5T		E > ctr	FDR-corrected *p*-value of 0.05	16
10	Lewis	2006	JoCN	T, A	Silent events	Silently decide whether the sound source was an animal or a tool	J	–	20	1.5T		T > A	α < 0.05, corrected	9
11	Lewis	2006	JoCN	T, A	Silent events	Silently decide whether the sound source was an animal or a tool	J	–	20	1.5T		A > T	α < 0.05, corrected	2
12	Lewis	2005	J Neurosci	T, A	Silent events	Silently categorize each stimulus as either a tool or an animal sound	J	–	20	1.5T		T > A	Whole-brain corrected significance level of < 0.05	9
13	Lewis	2005	J Neurosci	T, A	Silent events	Silently categorize each stimulus as either a tool or an animal sound	J	–	20	1.5T		A > T	Whole-brain corrected significance level of < 0.05	3
14	Girau	2001	JoCN	E	Noise	Say OK in response to every stimulus ∩ name the stimulus and say OK in response to control stimuli	J	–	12	PET		E > noise	*P* < 0.05 corrected for M.C.	5
15	Thierry	2003	Neuron	E	Beep and noise scrambled	Listen (and decide whether the stimulus refers to an animal) ∩ Listen (and decide whether the temporal succession of sounds could be considered as ordered or not)	J	V	12	PET		E > words	*P* < 0.08 corrected for M.C.	2
16	Engelien	2006	J Neural trans	E	Rest	Listen	L	–	6	PET	R	E > rest	*P* < 0.001 uncorrected	14
17	Specht	2003	NeuroImage	E	Rest	Listen	L	–	12	1.5T	R	E > rest	*P* < 0.05 corrected for M.C.	9
18	Galati	2008	NeuroImage	Act and non-Act	Fixation	Determine silently whether or not a human was directly involved in the production of the action sound	J	V	26	3T	R	Act and nAct-fix	*P* < 0.05 corrected for M.C.	26
19	Bidet-Caulet	2005	NeuroImage	A (footsteps)	Rest	Listen and indicate the motion direction of the walker	J	V	10	3T	R	Walking > rest	*P* < 0.01	
20	Doehrmann	2008	Neuropsychologia	A, T	Fixation	Press a button whenever a specific (different for each subject and pre-assigned prior to fMRI scanning) target sound was presented	J	V	15	1.5T		A > T	*p* < 0.05 (uncorrected)	5
21	Doehrmann	2008	Neuropsychologia	A, T	Fixation	Press a button whenever a specific (different for each subject and pre-assigned prior to fMRI scanning) target sound was presented	J	V	15	1.5T		T > A	*p* < 0.05 (uncorrected)	8
22	Maeder	2001	Neuroimage	E	Rest	Press a button in response to animal cries in the recognition task and press a button when the targets were presented at different locations in the localization task	J	V	18	1.5T		Rec > Loc	*P* < 0.001 (*T* score of 3.65)	14
23	Sharda	2012	Neuroscience	E, H, A	Rest	Decide whether the first sound was loud or soft	J	V	20	3T	R	A > rest	*P* < 0.05 FWE	35
24	Sharda	2012	Neuroscience	E, H, A	Rest	Decide whether the first sound was loud or soft	J	V	20	3T	R	E > rest	*P* < 0.05 FWE	38
25	Sharda	2012	Neuroscience	E, H, A	Rest	Decide whether the first sound was loud or soft	J	V	20	3T	R	H > rest	*P* < 0.05 FWE	29
26	Lewis	2004	Cer Cortex	E	Reversed sounds	Press a button if they could recognize, identify the sound, were uncertain, could not recognize the sound	J	V	24	1.5T		E Rec > Unrec	*P* < 0.05 corrected for M.C.	10
27	Bergerbest	2004	JoCN	E	Noise	Decide whether the sound was generated by an animal	J	V	14	1.5T		E > noise	*p* = 0.001, uncorrected	9
28	Fecteau	2004	Neuroimage	E,A,M,H	Scanner noise	Listening	L	–	15	1.5T		A > non-vocal sounds	*P* = 0.05, corrected for multiple comparisons across the brain, except for the STS, where a *P* = 0.001 (uncorrected) was used	1
29	Goll	2012	Neuroimage	A,T	Meaningless inverted sounds	Listening	L	–	22	3T		A > T	pb0.05, FWE-corrected for multiple comparisons	6
30	Goll	2012	Neuroimage	A,T	Meaningless inverted sounds	Listening	L	–	22	3T		T > A	pb0.05, FWE-corrected for multiple comparisons	9
31	Lewis	2012	Front in system neuros	E,A,M,H	Silent events	Listen (and press a button after the offset of the stimulus) ∩ Listen (and silently decide whether a human was involved in the sound)	J	V	31	3T	S	E > silence	Yielded a whole-brain correction at α < 0.05	2
32	Lewis	2012	Front in system neuros	E,A,M,H	Silent events	Press a button after the offset of the stimulus	J	V	12	3T	S	Scene like sound > silent events	Yielded a whole-brain correction at α < 0.05	3
33	Lewis	2011	JoCN	E,A,M,H	Silent events	Silently decide whether a human was involved in the sound	J	–	14	3T		H > AME	*P* < 0.05, corrected for M.C.	6
34	Lewis	2011	JoCN	E,A,M,H	Silent events	Silently decide whether a human was involved in the sound	J	–	14	3T		A > HME	*P* < 0.05, corrected for M.C.	2
35	Lewis	2011	JoCN	E,A,M,H	Silent events	Silently decide whether a human was involved in the sound	J	–	14	3T		M > HAE	*P* < 0.05, corrected for M.C.	4
36	Lewis	2011	JoCN	E,A,M,H	Silent events	Silently decide whether a human was involved in the sound	J	–	14	3T		E > HAM	*P* < 0.05, corrected for M.C.	7
37	Adams	2002	Neuroimage	E	Rest	Match sound to word	J	V	12	1.5T	R	E > rest	*P* < 0.01 uncorrected	23

*Types of stimuli, task and control task employed, silent listening vs. judgments (L or J), button-press conditions, rest vs. silent events (R-S) employed as control condition in the contrast image that was analyzed, number of subjects investigated, contrast used in the present analysis, significant level of the reported activations (threshold), and number of selected foci for the ALE meta-analysis*.

*The studies involved different categories of environmental sounds: E, environmental sounds; S, speech sounds; A, animal sounds; Obj, object-related sounds; Act, action-related sounds; T, tool-related; H, human sounds (e.g., coughing, laughing); and M, mechanical sounds (e.g., helicopter, water)*.

*M.C., multiple comparisons; Ml, meaningless sound: created by modifying a real sound; Loc, localization; Rec, Recognized; Unrec, Unrecognized*.

Based on these criteria, data from a total of 25 articles (including 22 fMRI and 3 PET studies) were entered into the study (see Table 1). In total, 37 experiments, i.e., lists of activation foci, were included in the first meta-analysis because 8 studies reported coordinates for more than one contrast. In this case, all contrasts were included in the analysis, since all of them reflected ES-related activations, e.g., in Lewis et al. (2006), coordinates from two contrasts (tools vs. animal and animal vs. tools) were reported, and we included both. Please note that this is a common procedure as can be found in previous ALE meta-analyses (for instance in Caspers et al., 2010; Tomasino et al., 2012, 2014). Taken together, the meta-analysis included data from 263 subjects and 627 activation foci.

#### Statistical procedure

A statistical map was generated using lists of x, y, and z coordinates after transferring these foci into MNI space (Lancaster et al., 2007). The meta-analysis was completed using the revised version (Eickhoff et al., 2009, 2012) of the GingerALE 2.1.1 software (brainmap.org) for coordinate-based meta-analysis of neuro-imaging results (Turkeltaub et al., 2002; Laird et al., 2005, 2009). Using the False Discovery Rate (FDR) with *q* = 0.01, the test was corrected for multiple comparisons (Laird et al., 2005, 2009; Eickhoff et al., 2009, 2012), and a minimum cluster size of 100 mm^3^ was set. The resulting areas were anatomically labeled by reference to probabilistic cytoarchitectonic maps of the human brain using the SPM Anatomy Toolbox (Eickhoff et al., 2005). Using a Maximum Probability Map (MPM), activations were assigned to the most probable histological area at their respective locations.

In meta-analysis 1, we identified the neural regions that were found to be consistently activated when listening to ES across multiple studies. In meta-analyses 2–5 (see below) we investigated how design choices might influence the activation observed in the list of the fMRI studies we evaluated.

In meta-analysis 2 we investigated first the effect of the *type of stimulus*. Many of the included studies reported contrasts related to different types of ES mixed together, some reported activations related to a specific category of stimuli [Action-related stimuli (Action, tools, human-related stimuli): Experiments n. 3, 5, 10, 12, 18, 19, 21, 25, 30, 33; Animal-related stimuli: Experiments n. 2, 4, 6, 11, 13, 20, 23, 28, 29, 34]. We directly contrasted the two types of stimuli.

In meta-analysis 3 we addressed the effect of the *type of control sound*. Some studies compared ES stimuli to silent stimuli, rest or fixation conditions [Experiments n. 1, 16–19, 23–25, 31, 32, 37], others compared ES stimuli to other control auditory sounds [Experiments n. 2–15, 20–22, 26–30, 33–36]. We directly contrasted the two types of control conditions. Using silent events vs. control sounds, we compared studies in which the active task (sound recognition) was compared to silent events (or resting) as control condition vs. studies in which the active task (sound recognition) was compared to control sounds (thus other sounds) as control condition.

In meta-analysis 4 we addressed the weight of the *type of task*: making a category judgment [Experiments n. 3–15, 18–27, 31–37] compared to passive listening [Experiments n. 1, 2, 16, 17, 28–30]. We directly contrasted the two types of control conditions.

In meta-analysis 5 we addressed the weight of the *type of response*: button press [Experiments n. 2–9, 15, 18–27, 31, 32, 37] vs. no button press/silent decision [Experiments n. 1, 3, 10–14, 16, 17, 28–30, 33–36]. The condition tasks involving button/no-button presses do not necessarily evidence motor cortex activations as the contrast includes also many studies like for example study 5 from Table 1 in which action-related sounds are compared to non-living-related sounds, but both categories (action and non-living) require a button press, and motor cortex activation has been subtracted out.

### Study 2: Neuropsychological study

#### Participants

##### Patients

###### Inclusion/exclusion criteria.

We included 7 neurosurgical patients meeting the following inclusion/exclusion criteria. Inclusion criteria were: a lesion involving areas included in the results of the meta-analysis, i.e., right and left temporo-insular-opercular cortex, being native Italian speakers, normal or corrected-to-normal vision and no history of psychiatric disease or drug abuse. Patients were excluded if they reported a hearing loss (as measured by the audiogram examination routinely performed before surgery), previous history of neurological problems or family history of developmental language problems or learning disabilities as well as inadequate speech comprehension, as they needed to understood the task and the instructions (for the tests included in their neuropsychological screening, see Table 2). Patients should not present with aphasia, as measured with standardized clinical tests (see Table 2). In particular, by excluding patients with aphasia and naming deficits we made sure that any impairment in ES recognition would not be related to language problems but reflect impaired ES processing. Lastly, among patients with lesions involving the right hemisphere we excluded those who had visuo-spatial/attentive deficits to make sure that any impairment in ES recognition would not be related to disorders of spatial attention (i.e., auditory) but reflect impaired ES processing.

**Table 2:** **Patients' neuropsychological profile**.

	**P1**	**P2**	**P3**	**P4**	**P5**	**P6**	**P7**
**Hemisphere**	**RH**	**RH**	**RH**	**RH**	**LH**	**LH**	**LH**
Lesion	STG+Ins+PostC	Ins+Rol	PreC+Ins*B.G.+T	PreC+PostC+Ins	PostC+PreC+IFG	F+Ins+Rol	STG+Rol
Volume (cc)	150.62	9.94	155.69	161.00	53.20	110.89	54.20
Age	49	69	49	63	44	36	66
Type	HGG	HGG	LGG	LGG	HGG	LGG	LGG
Years of schooling	13	18	13	13	13	18	5
Sex	F	M	F	M	M	M	F
Handedness	−100%	100%	100%	100%	100%	100%	100%
N.V. Intelligence	32/36	30/36	31/36	35/36	32/36	34/36	28/36
Comprehension	n.e	n.e	n.e	n.e	35,5/36	36/36	32/36
Naming nouns	n.e	n.e	n.e	n.e	29/30	30/30	28/30
Naming verbs	n.e	n.e	n.e	n.e	27/28	28/28	**22/28**
Fluency	n.e	n.e	n.e	n.e	**16**	31	31
ST memory	n.e	n.e	n.e	n.e	n.e	6/9	**2/9**
Oral praxis	n.e	n.e	n.e	n.e	20/20	20/20	20/20
IMA	n.e	n.e	n.e	n.e	72/72	72/72	71/72
Constructional apraxia	14/14	14/14	12/12	14/14	n.e	n.e	n.e
Visuo-spatial ability	54/54	54/54	**35/54**	54/54	n.e	n.e	n.e
Attention	n.e	24″	58″	54″	n.e	n.e	n.e
Visuo-spatial planning	n.e	10/10	10/10	9,5/10	n.e	n.e	n.e

*RH, right hemisphere; LH, left hemisphere; STG, superior temporal gyrus; Ins, Insula; Rol, Rolandic operculum; IFG, inferior frontal gyrus; Post/PreC, post-central/precentral, B.G., basal ganglia; HGG, High-grade glioma; LGG, low-grade glioma*.

*Pathological scores are shown in bold. Handedness [Edinburgh Handedness Inventory (Oldfield, 1971)]; N.V., Non-verbal intelligence [Raven's Colored Progressive Matrices (Basso et al., 1987)]; ST, verbal short-term digit span memory (Orsini et al., 1987); oral apraxia (Spinnler and Tognoni, 1987); IMA, ideomotor apraxia (De Renzi et al., 1980); visuo-spatial ability [star cancelation, (Wilson et al., 1987)]; visuo-spatial planning [clock test, (Mondini et al., 2011)]; constructional apraxia (Spinnler and Tognoni, 1987); attention [trial making test, B-A (Giovagnoli et al., 1996)]; Comprehension [Token test (De Renzi and Faglioni, 1978)]; verbal fluency (Novelli et al., 1996); noun and verb naming (Miceli et al., 1994)*.

*n.e., not executed*.

Seven right-handed neurosurgical patients (4M, 3F) (mean age 55.57 ± 12.47 years, and mean years of schooling 13.28 ± 4.34 years) were admitted to the local General Hospital some days before the beginning of the study. We tested patients' ES recognition ability before surgery. Each patient received a neuropsychological battery the day before the fMRI. The neuropsychological evaluation included tests assessing non-verbal intelligence, verbal short-term memory, praxis, visuo-spatial ability and planning, constructional apraxia, and language. All the patients performed these tasks successfully (See Table 2). Conventional T2-weighted MR imaging revealed low-grade lesions (82.17 ± 50.77, range: 9.94–161 mean cc in volume). The lesion overlap of all the patients showed that the lesion involved part of the left and right superior and MTG, temporal pole, hippocampus and parahippocampal area, insula, rolandic operculum, IFG (pars opercularis and triangularis), precentral gyrus and basal ganglia (see **Figure 2A**). The overlay plot of all the patients' lesions indicated the voxels most frequently damaged (see the bar code). The most frequent area (in bright green-yellow) corresponds to the hippocampus/insula/STG (see **Figure 2A**).

The study was approved by the Ethics Committee of our Institute and performed in accordance with the 1964 Declaration of Helsinki and subsequent amendments. The subjects' consent was obtained.

#### Environmental sound auditory confrontation naming task

##### Stimulus norming study

The primary goal of our norming study was to create a corpus of stimuli and responses, develop scoring criteria and determine a cut-off for the patients' Z scores. We used the original Marcell et al. (2000)'s corpus of stimuli (*N* = 120) of everyday, non-verbal digitized sounds belonging to many different categories such as sounds produced by animals, people, musical instruments, tools, signals, and fluids (Marcell et al., 2000) to conduct our own rating study for the Italian population, as there might be population-dependent differences in sound knowledge and frequency. ^*^.waw files were downloaded from their archive (http://marcellm.people.cofc.edu/confrontation%20sound%20naming/confront.htm) as 16-bit ^*^.WAV files with a sampling rate of 22,050 Hz.

The stimulus norming study included 20 monolingual native Italian speakers (8 F, 12 M; mean age 37.15 ± 11.24; age range 21–56; mean handedness 88.88, range 100–50; mean years of education 13.65 ± 3.77 years, range 8–18) with no history of neurological or auditory symptoms, different from the group involved in the fMRI study. We found a significant difference in age between patients and healthy controls [*t*_(25)_*=* 3.63, *p* < 0.001] but no education or gender effect [*t*_(25)_ = −0.35, *p* > 0.05 and *t*_(25)_ = −0.23, *p* > 0.05]. All participants gave their informed consent.

Following Marcell et al. (2000), the participants' primary task was to carefully listen to sounds and name the stimuli. Sounds were presented at a comfortable, preset loudness established through pilot testing. Each of the randomly ordered sounds was presented once, and participants were allowed 30 s to complete their identification. The tasks lasted about 45 min. Presentation® software (Version 9.9, Neurobehavioral Systems Inc., CA, USA) was used for auditory stimuli presentation. Answers were recorded by a PC and written down by the experimenter for later analysis. The experimenter then used these responses to establish the patients' scoring accuracy.

In order to determine the mean accuracy and evaluate the subjects' responses, we used the same criteria as used by Marcell et al. (2000). In particular, the following were scored as correct: synonyms, accurate descriptions of the sound, plurals, self-corrections. By contrast, lack of response or a “don't know” type of response and generalized superordinate descriptions of the item were scored as incorrect inaccurate descriptions of the sound. Furthermore, our rating study revealed that there were some sounds that were recognized by healthy controls as different from the responses reported in Marcell et al.'s study (Marcell et al., 2000). For example, our healthy participants recognized sounds like “explosion” as a “shot” (*N* = 5/20 subjects) or sounds like “frying food” as rain (*N* = 9 subjects), or sounds like typewriter (manual) as cash register (*N* = 7/20 subjects). For these items, if patients responded in a similar way as controls, we accepted their responses as correct. Last, there were some sounds that were not identified by healthy controls, such as cutting paper or water dripping (in both instances, 10/20 [50%] subjects did not recognize the sound) or a sonar (6/20 [33%] subjects did not recognize the sound). For these items, if patients responded in a similar way as controls, we accepted their responses as correct. Following these criteria, the participants correctly identified 87.83% ± 4.38 sounds (range 80–94.44). This result is very similar to the mean naming accuracy reported by Marcell et al. (2000) in their rating study (82.18 ± 22.67).

Thus, following the results obtained in our norming study, we selected 90 stimuli from the original corpus of stimuli of Marcell et al. (2000). The list of 90 stimuli used for the experiment had the following characteristics: 6.09 ± 0.90 mean familiarity, 3.19 ± 0.53 mean complexity, 3.92 ± 1.17 mean pleasantness, 2.47 ± 1.31 s mean duration, 82.18 ± 22.67 mean naming accuracy, and 6.06 ± 0.98 mean confidence in naming accuracy.

##### Task and procedure

Patients were asked to carefully listen to some sounds and name the stimuli (“Identify each sound as quickly and as accurately as you can”). We used the naming task similarly to Marcell et al.'s study. Sounds were presented at a comfortable, preset loudness established through pilot testing. Each of the randomly ordered sounds was presented once, and participants were allowed 30 s to complete their identification. Presentation® software (Version 9.9, Neurobehavioral Systems Inc., CA, USA) was used for stimuli presentation. Answers were recorded by a PC and written down by the experimenter for later analysis. Both the patients and healthy controls performed this task prior to the fMRI session. A typical testing session lasted 45 min.

##### Data analysis

Responses were analyzed by two independent raters. Accuracy was computed following the guidelines of Marcell et al. (2000) and according to the results of our own stimulus rating study (see below). For each patient we determined the Z score to calculate the number of patients whose performances were below the normal range (the reference group was healthy individuals). In addition, we performed a qualitative analysis of errors and labeled them as: semantically related to the target sound, auditorily related to the target sound (some sounds were both semantically and auditorily related to the target sound, in which case we coded them as semantically and auditorily related), unrelated and “I don't know,” and we expressed the total number of the different types of errors as % of the total errors. Last, we coded the errors according to the sound category by Marcell et al. (2000) (in their paper, a classification of sounds according to 27 categories can be found in Table 10).

### Study 3: Functional magnetic imaging (fMRI) study

The same ES auditory confrontation naming task with the same stimulus list described above was used during fMRI measurements involving the patients included in Study 2. Study 2 was a stimulus norming study including the original Marcell et al. (2000)'s corpus of stimuli (*N* = 120), whereas Study 3 included the final set of 90 stimuli. In the fMRI study, patients and healthy participants (see below) silently named the stimuli. We carefully instructed the subjects on how to perform the task. We asked them to listen carefully and name each stimulus, and at the end of the fMRI acquisition they would be asked some questions about each stimulus. Patients were highly motivated to perform the fMRI task as they knew that the fMRI maps are part of their clinical examination. More importantly, during fMRI acquisition we routinely performed online General Linear Model (GLM) analysis and continuously checked the activation and the BOLD signal correlation with the alternation of task and rest. If the GLM analysis showed that activation correlated significantly with the task, patients were performing the task appropriately. On the contrary, if no correlation emerged, we stopped the acquisition, talked to the patient and started the task again.

#### Healthy controls for the fMRI study

The patients' fMRI maps were compared with those of a control group consisting of 12 monolingual native Italian speakers (7 F, 5 M; mean age 35.75 ± 4.2 years old; age range 29–41; mean handedness 88.88 ± 17.88, range 100–50; mean education 16.5 ± 2.23 years, range 13–18). We found a significant difference age and education [*t*_(17)_ = 19.82, *p* < 0.001 and *t*_(17)_ = −3.2, *p* < 0.05] between patients and healthy controls, but not a gender effect [*t*_(17)_ = −0.41, *p* > 0.05].

All participants had normal or corrected-to-normal vision and no history of neurological illness, psychiatric disease, or drug abuse. Following Marcell et al. (2000), we checked that none of the healthy controls responded affirmatively to the self-report question, “To the best of your knowledge, do you have a hearing loss?” All gave their informed consent to participate in the study.

#### Task and procedure

The task started with an instruction (3 s). Subjects were asked to “carefully listen to the sounds and silently name the source of each sound as accurately and as quickly as possible.” During auditory stimulation a fixation cross was present on the screen. Blocks of ES recognition stimuli (*N* = 18, 15 s each) were alternated with baseline resting periods (*N* = 17, 15 s each, plus two additional resting blocks, one at the beginning of the run and the other at the end). In the baseline condition, a fixation cross (15 s) was presented between blocks and patients and controls were asked to relax. Each 15-s block included 5 stimuli, for a total of 90 stimuli. The same stimulus list used in the off-line pre-fMRI testing was presented during scanning. Presentation® software (Version 9.9, Neurobehavioral Systems Inc., CA, USA) was used for stimulus presentation and synchronization with the MR scanner. Participants listened to the stimuli via an Audio System (Resonance Technology).

#### fMRI data acquisition

A 3-T Philips Achieva whole-body scanner was used for both the patients and healthy controls to acquire T1-weighted anatomical images and functional images using a SENSE-Head-8 channel head coil and a custom-built head restrainer to minimize head movements. For both the patients and controls, functional images were obtained using a T2^*^-weighted echo-planar image (*N* = 222 EPI) sequence of the whole brain. The imaging parameters were as follows: repetition time, *TR* = 2500 ms; echo time, *TE* = 35 ms, field of view, *FOV* = 23 cm, acquisition matrix: 128 × 128, slice thickness: 3 mm with no gaps, 90° flip angle, voxel size: 1.8 × 1.8 × 3 mm; parallel imaging, SENSE = 2), and were preceded by 5 dummy images that allowed the MR scanner to reach a steady state.

For healthy controls, high-resolution anatomical images were acquired using a T1-weighted 3D magnetization-prepared, rapid acquisition gradient fast filed echo (T1W 3D TFE SENSE) pulse sequence (*TR* = 8.2 ms, *TE* = 3.76 ms, *FOV* = 24 cm, 190 transverse axial slices of 1 mm thickness, 8° flip angle, voxel size: 1 × 1 × 1 mm) lasting 8.8 min.

For patients, fMRI scanning was always performed before gadolinium injection and 6–10 days prior to craniotomy. In addition, high-resolution T2-weighted and post-gadolinium contrast T1-weighted anatomical MR images were acquired for use with the stereotactic surgical navigation system by using a T1-weighted 3D magnetization-prepared, rapid acquisition gradient-echo fast field echo (T1W_3D_TFE SENSE) pulse sequence (*TR* = 8.1007 ms, *TE* = 3.707 ms, *FOV* = 240.000 mm, 190 sagittal slices of 1 mm thickness, flip angle = 8°, voxel size: 1 × 1 × 1) and a T3-weighted 3D magnetization-prepared, rapid acquisition gradient-echo fast field echo (T2W_3D_TFE SENSE) pulse sequence (*TR* = 2500 ms, *TE* = 368.328 ms, *FOV* = 240.000 mm, 190 sagittal slices of 1 mm thickness, flip angle = 90°, voxel size: 1 × 1 × 1).

#### fMRI data processing and whole brain analysis

fMRI data pre-processing and statistical analysis were performed on UNIX workstations (Ubuntu 8.04 LTS, i386, http://www.ubuntu.com/) using MATLAB r2007b (The Mathworks Inc., Natick, MA/USA) and SPM5 (Statistical Parametric Mapping software, SPM; Wellcome Department of Imaging Neuroscience, London, UK). Dummy images were discharged before further image processing. Pre-processing included spatial realignment of the images to the reference volume of the time series, segmentation producing the parameter file used for normalization of functional data to a standard EPI template of the Montreal Neurological Institute template provided by SPM5, re-sampling to a voxel size of 2 × 2 × 2 mm, and spatial smoothing with a 6-mm FWHM Gaussian kernel to meet the statistical requirements of the General Linear Model and to compensate for residual macro-anatomical variations across subjects.

We checked that the movement parameters for all the patients and healthy controls were < 3 mm for translation and < 3 for rotation. We used the lesion masking image, i.e., a ROI image drawn on the patient's lesion in which the voxels are coded as 0 (tumor area) and 1 (healthy brain tissue). In the normalization procedure, we included the lesion masking image following Brett et al's technique (2001). This procedure allows to exclude the masked region (i.e., the lesion that would otherwise produce artifacts altering the normalization outcome) from normalization. Then, the normalization outcome was inspected carefully. In particular, three observers (B.T., D.S., and M.M.) independently compared the original and the normalized images and excluded any distortion phenomenon.

To delineate the network related to the ES recognition task, we modeled the alternating epochs by a simple boxcar reference vector. A general linear model for blocked designs was applied to each voxel of the functional data by modeling the activation and the baseline conditions for each subject and their temporal derivatives by means of reference waveforms which correspond to boxcar functions convolved with a homodynamic response function (Friston et al., 1995a,b). Furthermore, we included 6 additional regressors that modeled the head movement parameters obtained from the realignment procedure. Accordingly, a design matrix, which comprised contrast modeling alternating intervals of “activation” and “baseline” (resting), was defined. At a single subject level, specific effects were assessed by applying appropriate linear contrasts to the parameter estimates of the baseline and experimental conditions resulting in t-statistics for each voxel. For the single-subject first-level analysis, low-frequency signal drifts were filtered using a cut-off period of 128 s. These t-statistics were then transformed into Z-statistics constituting statistical parametric maps (SPM{Z}) of differences across experimental conditions and between experimental conditions and the baseline. SPM{Z} statistics were interpreted in light of the theory of probabilistic behavior of Gaussian random fields (Friston et al., 1995a,b).

With regard to second-level random effects analyses for both patients and healthy controls, contrast images obtained from individual participants were entered into a one-sample *t*-test to generate a SPM{T} indicative of significant activations specific for this contrast at the group level. For both patients' and controls' group we included age and education as covariate. We used a threshold of *P* < 0.05, corrected for multiple comparisons at the cluster level [using family-wise error (FWE)], with a height threshold at the voxel level of *P* < 0.001, uncorrected.

The following contrast images were calculated: first, we estimated the main effects of CONDITION (ES listening–baseline for the controls > task ES listening–baseline for the patients), then we performed a conjunction null analysis (and not a global null analysis) (Friston et al., 1999), showing the commonly activated network for both tasks (ES listening–baseline for the patients > ES listening–baseline for the controls) using a threshold of *p* < 0.05, corrected for multiple comparisons at the cluster level (using FWE), with a height threshold at the voxel level of *p* < 0.001, uncorrected. The anatomical interpretation of the functional imaging results was performed using the SPM Anatomy toolbox (Eickhoff et al., 2005).

## Results

### Study 1: Meta-analysis study of the reviewed fMRI studies about environmental sound processing

**Figure 1:**
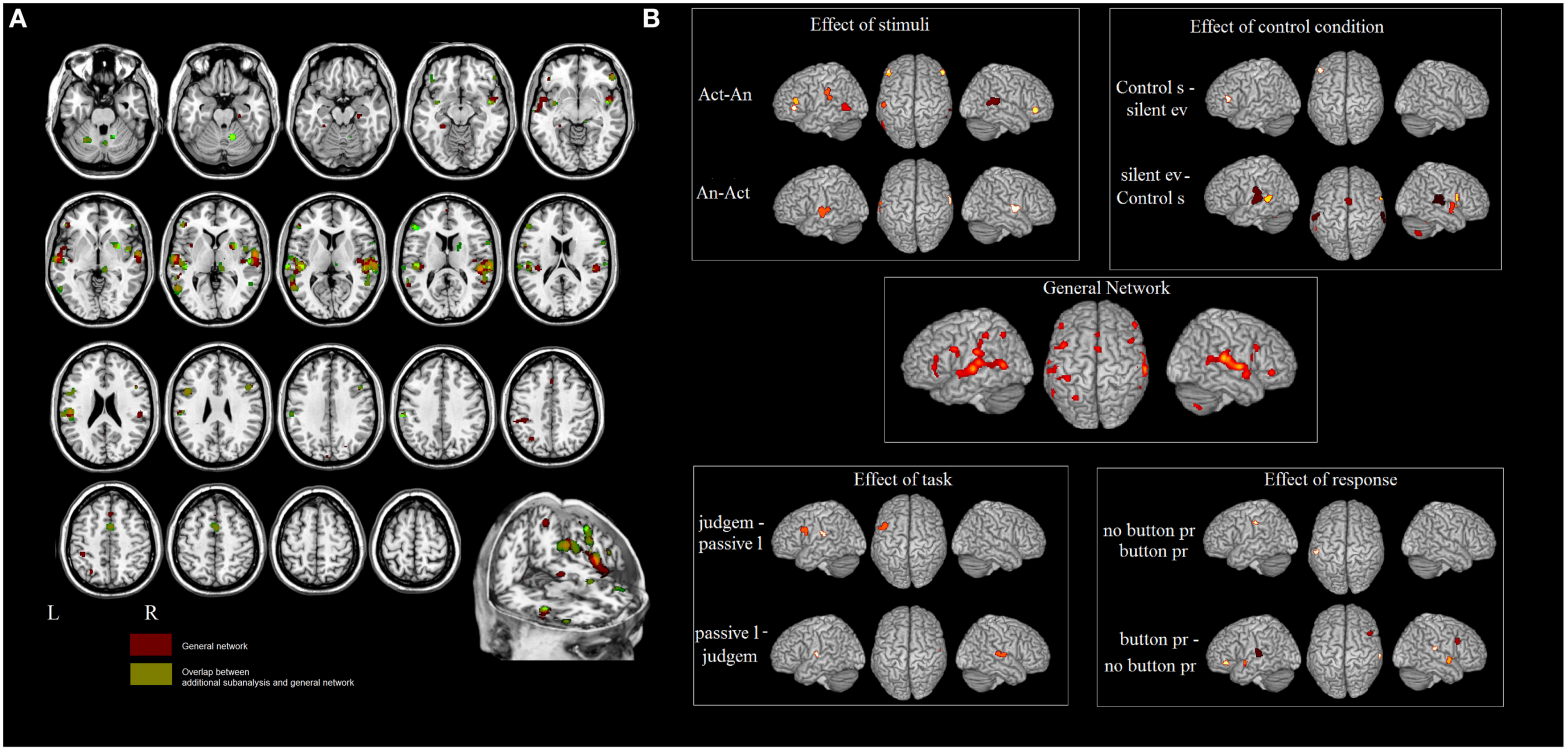
**(A)** Results of the ALE meta-analysis. The overlap between the general network and the networks found in meta-analyses 2–5 (in green). In red the areas not influenced by the external factors. **(B)** Influence of different design choices on the ES network.

The activation clusters resulting from meta-analysis 1 of all the reviewed studies comprised: (i) the right STG, extending to the MTG and Heschl's gyrus, the insula and the operculum [clusters 1, 2 and 4]; (ii) the left MTG extending to the STG and the supramarginal gyrus (SMG) and the insula [clusters 13 and 14]; (iii) the right [cluster 3] and the left [cluster 14] parahippocampal gyrus; (iv) the right IFG [clusters 5 and 6] and the left IFG including the pars orbitalis, triangularis and opercularis and the precentral area [clusters 16 and 17]; (v) the SMA [clusters 7 and 8]; (vi) the post-central, the inferior and superior parietal lobule [clusters 18 and 19]; (vi) the right putamen and thalamus [clusters 9 and 10]; and (vii) the right [clusters 11 and 12] and the left [clusters 20 and 21] cerebellum (see Table 3 and Figure 1)^1^.

**Table 3:** **Results of the ALE meta-analysis**.

**Custer**	**Region**	**Side**	**MNI**			**ALE Max**	**Cluster size**
			**x**	**y**	**Vox**		**Voxels**
**GENERAL NETWORK**							
1	Superior temporal gyrus	RH	50	−28	12	0.033	1311
	Middle temporal gyrus	RH	54	−52	6	0.017	
	Heschl's gyrus	RH	42	−18	8	0.017	
2	Superior temporal gyrus	RH	48	2	−10	0.021	220
	Insula	RH	46	2	−2	0.018	
3	Parahippocampal gyrus	RH	24	−22	−20	0.016	34
4	Rolandic operculum	RH	62	10	14	0.013	27
5	Inferior frontal gyrus (p. orbitalis)	RH	52	36	−8	0.021	84
6	Inferior frontal gyrus (p. opercularis)	RH	46	16	28	0.018	81
7	SMA	RH	2	4	48	0.019	109
8	SMA	RH	0	24	46	0.015	62
9	Putamen	RH	28	4	0	0.024	43
10	Thalamus	RH	12	−30	−2	0.015	78
11	Cerebellum	RH	22	−66	−52	0.022	92
12	Cerebellum	RH	14	−54	−24	0.023	62
13	Middle temporal gyrus	LH	−60	−14	0	0.031	1587
	Superior temporal gyrus	LH	−46	−26	8	0.026	
	Supramarginal gyrus	LH	−52	−22	22	0.026	
14	Parahippocampal gyrus	LH	−26	−38	−12	0.018	56
15	Insula	LH	−36	−2	−8	0.017	45
16	Inferior frontal gyrus (p. triangularis)	LH	−48	38	2	0.017	184
	Inferior frontal gyrus (p. orbitalis)	LH	−44	34	−8	0.012	
17	Inferior frontal gyrus (p. opercularis)	LH	−46	8	26	0.022	169
	Precentral gyrus (Area 44)	LH	−56	6	20	0.016	
18	Post-central gyrus	LH	−40	−36	46	0.019	110
	Inferior parietal lobule	LH	−52	−36	42	0.012	
19	Superior parietal lobule	LH	−28	−62	46	0.018	56
20	Cerebellum	LH	−26	−58	−28	0.016	53
21	Cerebellum	LH	−21	−66	−56	0.015	27
**SUBTRACTION ANALYSES**							
**Button press—No button press**							
1	Superior temporal gyrus	LH	−46	−28	10	–	171
2	Inferior frontal gyrus (pars triangularis)	RH	48.5	16	23	–	75
3	Cerebellum	RH	17.33	−53.67	−23	–	69
4	Insula	LH	−36	−2	−14	–	48
5	Insula	RH	42	0	−8	–	36
6	Putamen	RH	32	7	−1	–	36
7	Inferior frontal gyrus (pars orbitalis)	LH	−41	30	−13	–	31
8	Superior temporal gyrus	RH	64	−28	16	–	25
**Button press—No button press**							
1	Inferior parietal lobe	LH	−54	−28	36	–	26
**Listen only—Listen plus Judgment**							
1	Superior temporal gyrus	RH	56	−30	6	–	131
2	Heschl's gyrus	LH	−50	−14	6	–	35
**Listen plus Judgment—Listen only**							
1	Inferior frontal gyrus (pars opercularis)	LH	−56	8	18	–	159
2	Rolandic operculum	LH	−46	−28	14	–	26
**Control sounds—silent events or rest**							
1	Inferior frontal gyrus (pars triangularis)	LH	−42	32	12		77
**Silent events or rest—Control sounds**							
1	Superior temporal gyrus	LH	62.6	−24.4	9.6	–	446
2	Supramarginal gyrus	RH	−50	−16	20	–	430
3	Middle cingulate cortex	RH	2.04	4.24	47.8	–	168
4	Cerebellum	LH	21.9	−65.9	−51.7	–	143
5	Insula	RH	48	−2	−6	–	120
6	Cerebellum	RH	−26.9	−60.3	−28.3	–	106
7	Putamen	LH	24	10	−2	–	100
8	Cerebellum	LH	9.33	−54.6	−25.3	–	96
9	Middle temporal gyrus	LH	−62	−44	10	–	78
10	Precentral gyrus	LH	64	12	16	–	55
11	Cerebellum	LH	22	−50	−34	–	41
12	Cerebellum	RH	2.5	−64.2	−27	–	32
**Action related sounds—Animal sounds**							
1	Middle temporal gyrus	RH	56.09	−44.8	9.91	–	202
2	Middle temporal gyrus	RH	−56.21	−59.5	3.54	–	198
3	Supramarginal gyrus	LH	−58.8	−28.4	35.2	–	120
4	Inferior frontal gyrus (pars triangularis)	RH	−46.09	31.83	12.09	–	97
5	Inferior frontal gyrus (pars orbitalis)	RH	51.24	35.62	−3.33	–	94
6	Inferior frontal gyrus (pars triangularis)	LH	−49	36	5	–	40
**Animal sounds—Action related sounds**							
1	Superior temporal gyrus	LH	−64	−22	6	–	210
2	Superior temporal gyrus	RH	62.67	−9.33	4.67	–	174

*The general network involved in ES recognition and the results of the subtraction analyses are reported. Peaks of activation corrected above the threshold, MNI Coordinates (x, y, z) of maximum ALE value, and maximum ALE value of this cluster. All peaks are assigned to the most probable brain areas as revealed by the SPM Anatomy Toolbox (Eickhoff et al., 2005)*.

In meta-analysis 2 we investigated first the effect of the *type of stimulus*. Action- vs. animal- related stimuli activated the left and right MTG, the left SMG, the left pars triangularis of the IFG and the right pars orbitalis. Animal- vs. action- related stimuli activated the left and right STG (see Table 3 and Figure 1). As indicated in Figure 1, data show that part of the network related to ES processing is influenced by the “type of stimulus” factor.

In meta-analysis 3 we addressed the effect of the *type of control sound*. Some studies compared ES stimuli to silent stimuli, rest or fixation conditions, and others compared ES stimuli to other control auditory sounds. Control sounds vs. silent events or rest activated the IFG (pars triangularis). Silent events or rest vs. control sounds activated the right STG and the left MTG, the left SMG and the right precentral gyrus, the right insula, the right putamen, the middle cingulate cortex, and the cerebellum bilaterally (see Table 3 and Figure 1).

In meta-analysis 4 we addressed the weight of the *type of task*: category judgment vs. passive listening. Part of the activation found in the left IFG (pars opercularis) and left rolandic operculum was related to making a category judgment compared to passive listening. This contrast revealed activation in the right STG and the left Heschl's gyrus (see Table 3 and Figure 1).

In meta-analysis 5 we addressed the weight of the *type of response*: button press vs. no button press/silent decision. Part of the activation found in the right and left STG, the right and left insula, the right and left IFG (pars triangularis and orbitalis), the right putamen and the right cerebellum is related to button press. The contrast revealed activation in the left inferior parietal lobe only (see Table 3 and Figure 1).

To sum up, the networks found in meta-analyses 2–5 do not tap ES-related activations because, according to the logic of cognitive subtraction, this is “subtracted out” and the resultant map reflects the effect of an external variable (i.e., type of stimulus, or type of response) on the network.

Figure 1A shows the overlap between the general network and the networks found in meta-analyses 2–5 (in green). The areas that are not influenced by the effect of any external variable are shown in red. These included: the hippocampal area bilaterally, the right rolandic operculum, part of the STG bilaterally, and the left post-central area and superior parietal lobule.

### Study 2: Neuropsychological study on environmental sound recognition performance

**Figure 2:**
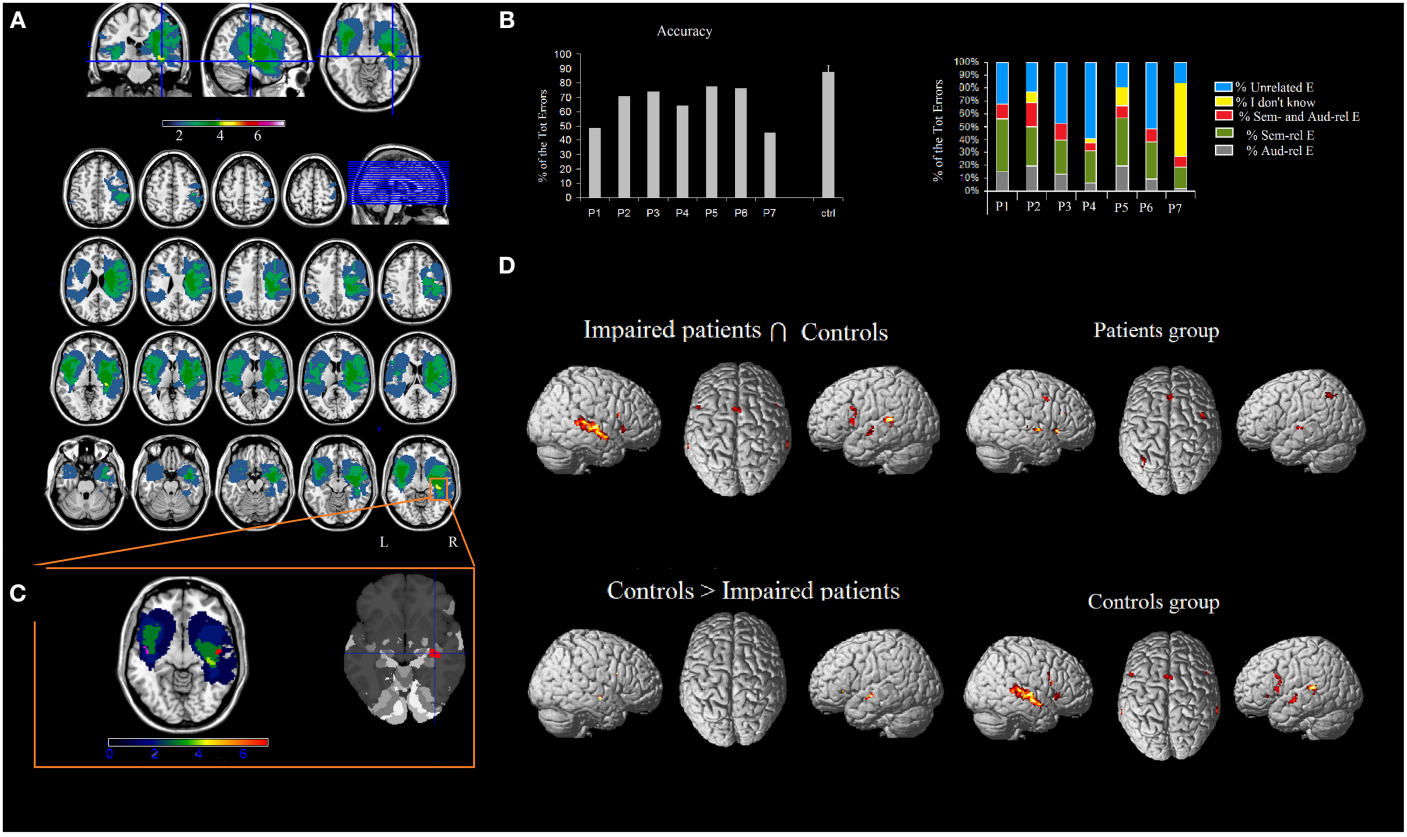
**(A)** Overlap of the patients' lesions in standard space. MRIcron software (http://www.mccauslandcenter.sc.edu/mricro/mricron/index.html) was used to draw the patients' lesions on their T1 and T2 MRI scans, creating the ROIs which were normalized to the MNI space using the “Clinical Tool box” (http://www.mccauslandcenter.sc.edu/CRNL/clinical-toolbox) for SPM8 (http://www.fil.ion.ucl.ac.uk/spm/software/spm8/). The results highlighted the areas of the brain that are related to the deficit (Karnath et al., 2004). The number of overlapping lesions is illustrated by different colors that code for increasing frequencies (as indicated in the bar code). **(B**) Patients' pathological performance (mean accuracy) and healthy controls' accuracy and patients' qualitative analysis of errors. **(C)** The most frequently lesioned area (in bright green-yellow) is the hippocampus/insula/superior temporal gyrus, as shown by the Anatomy toolbox. By using Marsbar (http://marsbar.sourceforge.net/), we drew two ROIs on the two clusters found in the left and in the right STG (which were less activated in patients than controls), shown respectively in pink and in red. The density bar shows that at least 4 out of 7 patients' lesions overlapped with the ROIs drawn on the STG. **(D)** Network of areas commonly activated in patients and controls and areas differentially recruited by controls vs. patients during ES recognition in addition to the network for ES processing in patients and controls. Activations were superimposed on a brain template provided by spm5.

Table 4 and Figure 2 show the patients' performances on the ES confrontation naming task. Patients scored below the normal range (as measured by Z-scores). Most of the patients' responses were not related to target sounds (35.94 ± 17.21%, see Figure 2 and Table 5) or were semantically related to target sounds (29.72 ± 8.73%). The other types of responses were: auditorily related (12.19 ± 6.66%), semantically and auditorily related (11.01 ± 4.20%), and “I don't know” answers (11.85 ± 20.72%) (unrecognized by patients but correctly identified by controls). Last, we coded the errors according to the sound categories by Marcell et al. (2000) (see their Table 10 for a classification of sounds according to 27 categories). We found that the 15.14% of the patients' errors involved musical instruments, 14.74% involved animal sounds, 37.45% involved other categories (e.g., transportation, nature, signals, accidents, weapons), and 32.67% involved actions/human sounds. Note that stimuli belonging to “musical instruments” and “animal sounds” are less numerous than those belonging to the “other” and “actions/human sounds” categories). For this reason, any further investigation of living vs. non-living related differences was not addressed.

**Table 4:** **Environmental sound recognition performance and qualitative error analysis in patients and healthy controls**.

**Patients**	**N. Correct responses**	**% Correct responses**	**Z score**	**% Aud-related Err**	**% Sem-related Err**	**% Sem-and Aud- related Err**	**% “I don't Know”**	**% Unrelated E**
P1	44/90	48.89	−8.87	15.22	41.3	10.9	0.00	32.61
P2	64/90	71.11	−3.81	19.23	30.8	19.2	7.69	23.08
P3	67/90	74.44	−3.05	13.04	26.1	13	0.00	47.83
P4	58/90	64.44	−5.33	6.25	25	6.25	3.13	59.38
P5	70/90	77.78	−2.29	20.00	40	10	15.00	20.00
P6	69/90	76.67	−2.54	9.52	28.6	9.52	0.00	52.38
P7	41/90	45.56	−9.63	2.04	16.3	8.16	57.14	16.33
Mean controls	79.05/90	87.84	–	–	–	–	–	–
Mean S.D. controls	3.95	4.38	–	–	–	–	–	–

**Table 5:** **Brain regions showing a significant increase in BOLD response for environmental sound listening in (i) *controls* > patients, and (ii) *patients* > controls, and common to both (∩)**.

**Region**	**Side**	**MNI**			**ALE Max**	**Cluster size**
		**x**	**y**	**Vox**		**Voxels**
**PATIENTS** > **CONTROLS**						
Anterior cingulate	M	−8	22	22	4.76	45
Inferior parietal lobule	LH	−36	−56	46	3.94	39
Thalamus	RH	16	−10	8	3.81	23
**CONTROLS** > **PATIENTS**						
Superior temporal gyrus	LH	−46	−12	−6	3.94	29
Superior temporal gyrus	RH	50	−10	−8	3.94	37
Inferior frontal gyrus (pars opercularis)	RH	40	8	28	3.59	24
Insula	LH	−32	22	2	3.36	21
**CONTROLS** **∩** **PATIENTS**						
Heschl's gyrus	RH	44	−22	10	4.30	761
Superior temporal gyrus	RH	54	−14	−2	4.01	
Superior temporal gyrus	LH	−54	−38	12	3.84	109
Middle temporal gyrus	LH	−66	−40	4	3.47	
Superior temporal gyrus	LH	−48	−12	−8	3.70	43
Insula	RH	38	24	0	3.83	60
Inferior frontal gyrus (pars opercularis)	LH	−52	12	28	3.53	33
Inferior frontal gyrus (pars opercularis)	RH	52	14	16	3.40	21
Inferior frontal gyrus (pars opercularis)	LH	−54	14	10	3.34	34
Supplementary motor area (SMA)	M	2	6	56	3.49	47

*For each region of activation, the coordinates in MNI space are provided with reference to the maximally activated voxel within an area of activation, as indicated by the highest Z-value (P < 0.05, corrected for multiple comparisons at the cluster level, height threshold P < 0.001, uncorrected). LH/RH, left/right hemisphere; M, medial*.

### Study 3: fMRI investigation

The areas showing a different activation in patients vs. controls (controls > patients) were: (i) the right STG, (ii) the left STG, (iii) the right IFG (pars opercularis), and (iv) the left insula extending to the IFG (pars triangularis) (see Figure 2 and Table 5). As to differences in activation across the STG, by using Marsbar (http://marsbar.sourceforge.net/), we drew two ROIs on the two clusters found in the left and the right STG (which were less activated in patients than controls), shown respectively in pink and in red in Figure 2B. We overlapped all the ROIs of the lesions checking the density bar showing how many patients had a lesion overlapping with the two ROIs. The map showed that the red ROI (right STG) overlapped with about 2 of the RH lesions, and that the pink ROI (left STG) overlapped with about 2 of the LH lesions. Taken together, these data suggest that the two ROIs on the two clusters found in the left and the right STG overlapped at least with 4 of the 7 lesions, indicating that the lack of activation in the STG is related to brain damage.

The reverse contrast (patients > controls) revealed a higher activation in patients vs. the control group, in the anterior cingulate, the right thalamus and the left inferior parietal lobule.

The functional areas whose activation was comparable to that of controls as revealed by the conjunction analysis (sound listening > baseline in patients > sound listening > baseline in controls) included: (i) the right Heschl's gyrus, extending to the STG, (ii) the left STG, extending to the MTG, (iii) the right IFG (pars opercularis), (iv) the left IFG (pars opercularis), (v) the SMA bilaterally, and (vi) the right insula (see Figure 2 and Table 5)^2^.

### Overlap of the ALE map with the fMRI map of patients and controls

**Figure 3:**
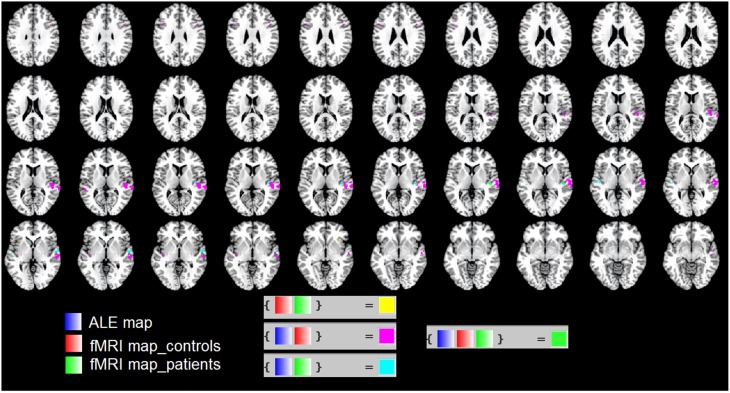
**We used the “Logical Overlays” function in Mango (http://ric.uthscsa.edu/mango/)**. We overlapped the ALE map (in blue) with the fMRI map of our patients (in green) and that of healthy controls (in red). Different combination of overlaps were included, e.g., fMRI control and fMRI patients; ALE map and fMRI controls. In particular, in green the overlap of the three maps in the STS.

In Figure 3 we used the “Logical Overlays” function in Mango (http://ric.uthscsa.edu/mango/). We overlapped the ALE map (in blue) with the fMRI map of our patients (in green) and that of healthy controls (in red). Different combination of overlaps were included, e.g., fMRI control and fMRI patients; ALE map and fMRI controls. As shown in Figure 3, the three maps overlap in the STS. This is consistent with the less activation in the STS found in patients vs. controls (see the two ROIs shown in Figure 2C).

## Discussion

In the present multimodal study we used a new approach combining different techniques and looking for converging evidence from multiple sources to explore the neuroanatomy of ES recognition.

The ALE meta-analysis delineated the core set of regions involved in ES as evidenced by fMRI literature. This analysis revealed the network of areas supporting ES recognition. We next showed that not all of the clusters truly reflect ES processing and how design choices, e.g., type of stimulus, type of task, type of control condition, might have influenced the activation observed in the fMRI studies we evaluated.

The hippocampal area bilaterally, the right rolandic operculum, part of the STG bilaterally, and the left post-central area and superior parietal lobule were not influenced by any of the factors which might influence the ES network. Part of this region (i.e., hippocampus, rolandic operculum, and STG/MTG bilaterally) was found to be most frequently lesioned in our patient sample.

The pathological performance of neurosurgical patients showed that areas of the ES network have a causal role in ES processing, since a lesion in those areas caused a deficit in ES recognition. Patients had a normal performance on the neuropsychological screening. In particular, by excluding patients with aphasia and naming deficits we made sure that any impairment in ES recognition would not be related to language problems but reflect impaired ES processing.

These results indicate that a deficit of ES recognition does not arise exclusively following lesions to the right hemisphere or left hemisphere. So far, no precise anatomical locations have been correlated with auditory agnosia (Lewis et al., 2004). Our data, thus, add new information to the ES recognition related literature, showing that both the left and the right hemisphere, if damaged, can cause a deficit in ES recognition.

One crucial area involved in ES is the STG/MTG. Although the meta-analysis showed that part of the left and right STG/MTG activation during ES processing might be in part related to design choices, this area was one of the most frequently lesioned areas in our patient sample, thus highlighting its causal role in ES processing. The planum temporale is the auditory association cortex and it represents the first (input and processing) node (or computational hub, Griffiths and Warren, 2002) of the network involved in segregating the components of the acoustic stimulus and matching these components with learned spectrotemporal representations. The information is then gated to higher-order cortical areas for further processing (Griffiths and Warren, 2002). The STG activation has been reported as reflecting the input stages of ES processing (Lewis et al., 2004). In our fMRI study, right STG activation was found in patients and controls. This suggests that these areas were still actively functional in patients, too. However, the controls > patients comparison revealed that a greater activation of a sub-part of the STG in controls vs. patients, meaning that patients lacked activation in a crucial sector of the STG. The two ROIs on the two clusters found in the left and the right STG overlapped at least with 4 out of 7 patients lesions, indicating that the lack of STG activation found for patients is related to brain damage. Several authors found bilateral activations in the STG during ES processing, with a larger region of activation in the right STG (Bergerbest et al., 2004). The right STG posterior to the primary auditory cortex has been proposed as a node of the “neural semantic detector model” describing semantic memory for non-verbal sounds (Kraut et al., 2006). Of course, as evidenced by the ES recognition model (Lewis et al., 2004), too, the information resulting from the described processing steps needs the intervention of the semantic system, which is lateralized to the LH. Accordingly, the meta-analysis showed that studies requiring active judgment or categorization as compared to those requiring passive listening additionally activated the left IFG and the left rolandic operculum.

Our ALE meta-analysis included the right and the left parahippocampal gyrus as it does the temporal part of the lesion map. Its role in sound recognition might be related to the localization of sounds or the mental spatial imagery of source sound localization. Some authors suggested that the activation of this area possibly reflects the “imageability” of sounds (Sharda and Singh, 2012). See also (Engel et al., 2009).

ES processing requires allocating auditory attention/memory to the input sounds. Accordingly, the right IFG has been related to auditory working memory (Zatorre et al., 1994; Zatorre, 2001), or to allocating auditory attention (Lipschutz et al., 2002; Binder et al., 2004). Interestingly, it has been shown that auditory verbal hallucinations predominantly activate the right IFG (Sommer et al., 2008). In our fMRI study, the direct controls > patients comparison revealed that a part of the right IFG was more activated in controls than in patients, meaning that impaired patients lacked activation in a crucial sector of the IFG. It has been suggested that ES is polymodal in nature and the IFG bilaterally is responsible for integrating polymodal object representations with concepts in semantic memory. Interestingly, we found that activation in different sectors of the IFG was related to many design choices. Only the right pars triangularis truly reflected activation related to ES processing. This region, together with the insula, is an area that was frequently lesioned in our patients. It is part of a finely tuned attentional network which selects information from the continuous flow of auditory signals and triggers communication and balance between the RH and LH according to the nature of the stimulus (Habib et al., 1995). The insula has numerous direct connections with the auditory cortex (Adams and Janata, 2002; Bamiou et al., 2003). A comparison of a patient with total agnosia following bilateral insular damage (Habib et al., 1995) with a case with no agnosia following left insula–thalamocortical projection damage (Hyman and Tranel, 1989) is indicative of the essential role of the bilateral insula in auditory stimuli pre-processing.

Since ES recognition is both a top-down and bottom-up driven process, it presupposes an interaction between many areas in the brain. And it also presupposes an involvement of the fiber tracts. In our patients, the lesions might also be interpreted in terms of damage to the fiber tracts. Indeed, many of the clusters discussed here are interconnected with the input nodes of the temporal cortex. Accordingly, in the temporal-parietal lobe area, the activation found in the ALE map, including the left precentral/post-central gyrus/supramarginal gyrus, was related to the type of stimulus. We found that action- vs. animal-related stimuli activated the left supramarginal gyrus. It is known that perceptual processing and semantic processing interact to represent ES. Thus, these activations might be related to the action/human sound category processing. With regard to action-related verbs and phrases, it has been shown that imagery of the verbal context could be responsible for activation in sensorimotor areas (e.g., Tomasino and Rumiati, 2013a,b).

## Conclusion

ES recognition is dependent on a bilateral network of areas in the temporal, inferior frontal basal ganglia, and areas of the pre- and post-central gyrus, as shown by the ALE meta-analysis. We showed that some of these clusters of activation truly reflect ES processing, whereas others are related to design choices.

The hippocampal area bilaterally, the right rolandic operculum, part of the STG bilaterally, and the left post-central area and superior parietal lobule were not influenced by any of the factors which might influence the ES network. In addition, the lesion map evidenced areas that are necessary for ES processing, namely the hippocampus, STG/MTS area and the rolandic operculum, which might be deregulated in activation as compared to healthy controls.

### Conflict of interest statement

The authors declare that the research was conducted in the absence of any commercial or financial relationships that could be construed as a potential conflict of interest.

## Footnotes

^1^All the studies we included reported activations surviving corrections for multiple comparisons except some (study 2, 16, 19, 20, 22, 27, and 37). We re-run the analysis by excluding these studies. We confirm that the same network of activation was found.

^2^Age and education did not influence the ES related network, as by including these two variables as covariate in our analysis, the same network was found for both patients' and controls' group.
